# Validation and Cultural Adaptability of the MOBAK Test Battery for Assessing Fundamental Motor Skills in Chinese Children Aged 3–12 Years

**DOI:** 10.3390/bs16040534

**Published:** 2026-04-02

**Authors:** Jingjie Zhang, Ke Ning, Bingjun Wan, Hongmiao Chen, Chen Wang, Yue Ye, Hongyou Liu

**Affiliations:** 1 School of Physical Education, Shaanxi Normal University, Xi’an 710119, China; 17605965053@163.com (J.Z.); bingjunw55@snnu.edu.cn (B.W.); chanceus@163.com (C.W.); 2 School of Physical Education, Huaqiao University, Xiamen 361021, China; chinacmm@hqu.edu.cn; 3 School of Sport Science, Beijing Sport University, Beijing 100091, China; yueye1030@163.com; 4 Research Center of Sports Performance Analysis, School of Physical Education & Sports Science, South China Normal University, Guangzhou 510006, China

**Keywords:** fundamental motor skills, child development, psychometrics, validation study

## Abstract

Accurate assessment of children’s fundamental motor skills (FMS) is crucial for promoting lifelong healthy development and formulating effective physical education policies. However, China currently lacks standardized assessment tools that cover the entire age range from 3 to 12 years and have undergone thorough cultural adaptation. This study aimed to evaluate the psychometric properties and cultural adaptability of the MOBAK assessment tool in measuring FMS in Chinese children aged 3 to 12 years. A total of 1200 Chinese children from four regions of China participated in the study, including 623 boys (52%) and 577 girls (48%). The MOBAK tool was used to assess FMS across different age groups, focusing on two dimensions: object movement (e.g., throwing, catching, bouncing, and dribbling) and self-movement (e.g., balancing, rolling, jumping, and running). The study evaluated psychometric properties, including reliability and validity. Results indicate that MOBAK demonstrates excellent psychometric characteristics: (1) Good item discrimination (all CR values *p* < 0.001), with an appropriate difficulty index (0.51–0.67); (2) Extremely high reliability, manifested by high internal consistency (α > 0.80), high test–retest stability, and high inter-rater consistency (ICC > 0.90); (3) Robust construct validity, supported by exploratory and confirmatory factor analyses, which consistently confirmed the hypothesized two-factor model and had excellent fit indicators (CFI/TLI > 0.90, RMSEA/SRMR < 0.08). The MOBAK battery demonstrates strong psychometric properties and cultural validity in the Chinese context for reliably assessing FMS in children aged 3–12 years. These findings provide a foundation for future cross-cultural comparisons and validation studies in other populations.

## 1. Introduction

Fundamental motor skills (FMS) represent essential movement patterns that emerge during childhood and serve as building blocks for complex motor behaviours, physical fitness development, and overall health outcomes. These skills play a pivotal role in children’s physical, cognitive, and social development trajectories (Bretz et al., 2022). With the increasing global emphasis on early childhood development and health promotion, assessing fundamental motor skills (FMS) has become a key research focus in exercise science, education, and public health (Lopes et al., 2021). Data from the WHO shows that over 80% of adolescents worldwide fail to meet the recommended daily level of physical activity (Guthold et al., 2020). In China, the situation is even worse—about 86% of children and adolescents are not very active (Wang et al., 2024). A recent large-scale cross-sectional study covering 14 provinces in China and involving 11,480 children aged 3 to 10 further revealed that the proficiency of basic motor skills among Chinese children is generally below 60%, and there is a significant developmental delay. Among them, the development of mobility skills and manipulation skills is unbalanced, and the failure rate of action components is relatively high (Xu et al., 2023). In addition, the prevalence rate of Developmental Coordination Disorder (DCD) among Chinese children is approximately 8.3%, and the detection rate among school-age children in Shanghai is 9.2% (Xue et al., 2024). These data highlight the urgency of systematically assessing and monitoring basic motor skills in Chinese children.

Accurate evaluation of children’s FMS is essential for understanding and fostering their motor development. In preschoolers, both quantitative and qualitative assessments are necessary, and quantitative methods measure aspects such as distance, speed, time, or frequency, while qualitative approaches evaluate how children perform movements, using criteria that reflect developmental patterns and skill components. To support scientific evaluation and effective intervention, it is vital to develop culturally appropriate, reliable, and valid tools for assessing FMS in young children (Clark & Whitall, 1989; C. Herrmann et al., 2019b). According to the theory of dynamic systems, the development of motion is not a predetermined or phased process, but rather the result of continuous interaction among individuals, the environment and task constraints (Thelen & Smith, 1994), In this framework, children’s motor abilities are shaped by specific “affordances” and demands in their culture and physical environment—including physical education programs, traditional games, and extracurricular activities (González-Víllora et al., 2019). This perspective highlights the significance of cultural sensitivity assessment tools, namely those capable of capturing the diverse sports experiences of different groups of people. If an assessment tool is developed and standardized within a single cultural context, it may fail to fully reflect the sports abilities of children from other cultural backgrounds, thereby leading to assessment errors (Greenholtz, 2005).

Existing research on FMS assessment tools has primarily focused on several key areas: (1) cultural adaptability. With the global promotion of FMS assessment tools, scholars have focused on how to improve them through localization to maintain consistency and reliability and validity across different cultural contexts; (2) validity and reliability verification, using rigorous statistical analysis methods to test the scientific validity of assessment tools. In recent years, numerous FMS assessment tools have been developed and widely applied globally. However, many tools prioritize outcomes over the process of movement execution (Gard & Rösblad, 2009). The most widely used tools include the Test of Gross Motor Development (TGMD) (Webster & Ulrich, 2017), the Körperkoordinationstest für Kinder (KTK) (Iivonen et al., 2016), and the Bruininks-Oseretsky Test of Motor Proficiency, Second Edition (BOT-2) (Beitel & Mead, 1980). While TGMD-3 demonstrates strong psychometric properties in Western populations, certain culture-specific items (e.g., baseball batting, American football catching) may not reflect the typical motor experiences of Chinese children. The physical development environment for Chinese children bears distinct cultural characteristics. For instance, traditional activities such as skipping rope, martial arts, and gymnastics occupy a significant place in both daily physical education and extracurricular activities, potentially compromising their cultural validity. The KTK offers a standardized and efficient assessment of balance and coordination, but does not evaluate object control skills like throwing or dribbling, limiting its comprehensiveness. BOT-2 provides a broad and diagnostic assessment of motor proficiency but involves prolonged testing procedures and requires specialized equipment. Currently, most existing tools are designed for specific age groups (e.g., preschool or post-school age) and lack a longitudinal assessment system for the entire age range of 3–12 years.

The MOBAK assessment tool is based on a two-factor model and has demonstrated high reliability and validity in European contexts. Compared to existing tools, MOBAK evaluates both object movement (object control) and self-movement (body movement), and compensates for the common omission of stability skills through the inclusion of balance-related tasks. This design offers a more comprehensive assessment of FMS (C. Herrmann et al., 2019a). MOBAK fundamentally circumvents the bias inherent in culturally specific tasks like the ‘baseball swing’ found in assessments such as TGMD-3 through its functional task design, incorporating elements like “bouncing” and “throwing”. This focus on fundamental movement objectives ensures the evaluation centres on children’s core motor abilities rather than their exposure to specific sports, thereby enhancing the comparability of results across diverse cultural contexts. By contrast, while KTK maintains cultural neutrality in assessing physical coordination, it entirely omits the evaluation of object control skills, presenting a significant dimensional deficiency. Furthermore, MOBAK’s modular architecture (covering four developmental stages: KG, 1–2, 3–4, and 5–6) enables seamless longitudinal tracking of children aged 3–12. This resolves the coverage gap in the upper primary school years present in TGMD-3. MOBAK’s equipment is straightforward and readily standardized for implementation within routine school settings. It also compensates for the BOT-2’s broad age coverage but overly complex and burdensome testing process, which hinders its use for large-scale, continuous group monitoring. MOBAK overcame some of the limitations of these tests. This positions MOBAK as a complementary approach to the clinical diagnostic orientation of BOT-2 and the process-oriented assessment of TGMD-3, offering a practical and functional alternative suitable for school-based settings.

In recent years, China has made certain progress in the development of domestic FMS assessment tools. For instance, the Children’s Basic Motor Skills Test (CFMST) has been developed and validated for preschool children (Ke et al., 2026). China still lacks assessment tools that cover the entire age range of 3–12 years old, have undergone strict reliability and validity tests, and are internationally comparable.

Since its development, the MOBAK assessment tool has been validated and applied across multiple countries and regions. Herrmann conducted the first systematic validation of MOBAK-1–2 two-factor structure among primary school children in Germany and Switzerland, confirming its sound psychometric properties and laying the groundwork for subsequent cross-national research. Building upon this, Wälti et al. (2022) conducted a large-scale cross-sectional study involving children aged 6 to 8 years across ten European countries. This further confirmed the MOBAK tools’ applicability in cross-cultural contexts. Findings revealed significant variations in fundamental motor skills among children across nations, closely correlated with age, gender, BMI, and participation in extracurricular physical activities (Wälti et al., 2022). Furthermore, some research applied the MOBAK-1–2, 3–4, 5–6 test among Chilean schoolchildren(Students with and without special educational needs), not only validating the tool’s effectiveness within a Latin American cultural context but also revealing a significant association between motor ability and adherence to physical activity recommendations (Martinez-Lopez et al., 2024; Linares-Guzman et al., 2026; Robles-Campos et al., 2025). Collectively, these studies demonstrate that the MOBAK tool exhibits stable construct validity and measurement consistency across diverse cultural, geographical, and educational settings.

The present study aims to evaluate the cultural validity of the MOBAK assessment tool in Chinese children aged 3–12 years. Specifically, we focus on structural equivalence—that is, whether the two-factor model (object movement and self-movement) originally developed in European samples can be replicated in the Chinese context. We hypothesize that: (1) the two-factor structure will be confirmed in Chinese children; (2) the MOBAK items will demonstrate adequate reliability (internal consistency, test–retest, inter-rater) and appropriate item properties (difficulty and discrimination); and (3) the factor structure will demonstrate sstructural equivalence with previous findings from European samples, providing initial evidence for cross-cultural validity. By offering the first empirical evidence on the psychometric properties of MOBAK in China, this study addresses the current lack of standardized FMS assessment tools covering the entire 3–12 age range and establishes a foundation for future research on measurement invariance across cultures.

## 2. Methods

### 2.1. Participant Sample

To ensure the scientific rigour and statistical adequacy of our research design, we conducted a Monte Carlo (Mplus Version 8.10, Muthén & Muthén, Los Angeles, CA, USA) simulation before formal data analysis. The simulation was based on a two-factor confirmatory factor analysis model with eight observed variables. All standardized factor loadings and inter-factor correlations were preset to 0.50. Using a sample size of 200 across 1000 repetitions, the simulation demonstrated excellent statistical properties.

All iterations converged successfully, with the model fit indices meeting ideal criteria (χ^2^/df = 1.026, RMSEA = 0.017, SRMR = 0.039). Parameter estimation was highly accurate: the mean estimates for factor loadings (0.4949–0.5028) and factor correlation (0.4941) closely approximated the preset value of 0.50, with a minimal mean absolute deviation of 0.003. Standard error estimates were also precise (average = 0.091). Statistical power was sufficient, ranging from 99.9% to 100% for factor loadings and 97.7% for the factor correlation. Furthermore, the 95% confidence interval coverage for most parameters (92.8–95.9%) approached theoretical expectations. This simulation confirms that a sample size of 200 provides accurate parameter estimates and sufficient power for the subsequent confirmatory factor analysis.

This study employed stratified cluster sampling to recruit a diverse sample of Chinese children from varied geographical and socio-economic backgrounds. Four provinces—Zhejiang, Shaanxi, Guangdong, and Neimenggu—were selected to represent the East, West, South and North regions of China, ensuring broad regional representation.

Children were excluded from the study if they met any of the following criteria: (1) diagnosed mental or physical impairments (e.g., intellectual disability, cerebral palsy, autism spectrum disorder) as reported by parents or documented in school medical records; (2) physical injuries at the time of testing that would prevent safe participation in the motor tasks; or (3) inability to understand or follow simple verbal instructions, as determined by the class teacher. Importantly, children with mild motor difficulties (e.g., suspected developmental coordination disorder or below-average motor performance) were not excluded, as the study aimed to capture the full spectrum of motor competence in the general population. Exclusion status was determined through a combination of parental report, school medical records, and consultation with class teachers before data collection. Initially, 1400 children were selected, and informed consent forms were distributed to their parents; 1295 forms were returned. After excluding cases with incomplete key data, the final sample comprised 1200 children aged 3–12 years (623 boys, 577 girls; see Table 1). All procedures were approved by the university ethics committee, and written parental consent and child verbal assent were obtained before data collection.

### 2.2. Assessment Tools

The MOBAK assessment system, developed by C. Herrmann et al. (2015) in Switzerland, was employed in this study. MOBAK systematically measures children’s motor performance in everyday physical activities by focusing on two core dimensions: object movement (e.g., throwing, catching, bouncing, dribbling) and self-movement (e.g., balancing, rolling, jumping, running), which together form the framework of its two-factor model. Within this model, stability (e.g., balancing) is categorized under self-movement. These abilities reflect children’s performance in self-body control and spatial movement, laying a crucial foundation for the execution of complex motor tasks and serving as a key dimension for assessing children’s overall motor abilities (Bolger et al., 2021; C. Herrmann et al., 2018).

### 2.3. Procedure

Rigorous standardized protocols were implemented to ensure data quality and measurement reliability. Cultural and linguistic adaptation of the MOBAK instrument (which was developed and validated by the Department of Sport, Exercise and Health at the University of Basel (Basel, Switzerland)was conducted through systematic forward and backward translation procedures. The specific process included the following steps: Firstly, a PhD in Education and two Master’s degree holders in Physical Education conducted a cultural context-specific localization translation of the original evaluation content to align it with Chinese cultural and semantic characteristics. Subsequently, the translated content was back-translated by two English language experts to verify the equivalence and semantic consistency of the translation. Finally, based on Chinese language conventions and cultural background, the Chinese version was compared with the original English version and the reverse-translated version to assess and revise the content validity. Five experts in relevant fields assessed the content validity index: I-CVI = 1 (Ke et al., 2023), resulting in the final Chinese version of the assessment scale.

To ensure consistency and standardization of the assessment methods, the research team provided systematic and rigorous standard operating procedure (SOP) training for assessors(All assessors must hold at least a bachelor’s degree in physical education or a related field). This includes theoretical knowledge, standardized action demonstrations, and detailed explanations of scoring criteria. Evaluators are required to demonstrate the 8 core motor skills in MOBAK proficiently and by established standards, and pass a series of assessments to validate their operational capabilities and scoring accuracy. This ensures that all testers adhere to uniformity and standardization during actual testing, thereby guaranteeing the reliability and validity of the assessment results.

Testing equipment and technical preparations were strictly conducted by the MOBAK evaluation standards, with all required equipment undergoing thorough technical calibration and configuration. All equipment required for the assessment, including rulers, tape, balls, balance beams, etc., was arranged by the standard configuration specified in the MOBAK manual to ensure the controllability of the testing environment and the reliability of data collection (Table 2). The use and placement of equipment strictly followed the principles of measurement error control to ensure the internal consistency and reliability of the assessment data and the repeatability of the results.

Assessments were scheduled between 8:30 and 11:30 AM and 3:00–4:30 PM. To minimize anxiety, a familiar teacher or assistant escorted children to the testing area (e.g., playground or sports hall). A trained assessor, blinded to the child’s prior performance, conducted individual assessments while other children waited in supervised areas. At the start of the test, evaluators described the test content and provided a single demonstration. Children then performed the test according to the MOBAK guidelines. Each participant had six attempts (throwing and catching), with the following scoring: 0–2 successful attempts scored 0 points, 3–4 successful attempts scored 1 point, and 5–6 successful attempts scored 2 points. For another set of tests (bouncing, dribbling, balancing, rolling, jumping, and running), each child had two attempts, with the following results: two failures scored 0 points, one successful attempt scored 1 point, and two successful attempts scored 2 points. The testing process includes short breaks between tasks to prevent fatigue. The on-site atmosphere is encouraging and non-competitive, and assessors provide standardized, neutral evaluations.

### 2.4. Statistical Analysis

All statistical analyses were performed using IBM SPSS Statistics 26.0 and Mplus 8.3. Descriptive statistics were presented in “mean ± standard deviation”. Content validity, according to the authoritative criteria established by Polit et al. (2007), when the expert panel comprises five members: The I-CVI should not be less than 0.99; a value of 1.00 indicates that the item possesses excellent content validity (Polit et al., 2007). Item analysis included the calculation of the Difficulty Index and Discrimination Index. The Difficulty Index was defined as the ratio of the average score for each item to its maximum possible score, with lower values indicating greater difficulty and higher values indicating easier tasks (Hopkins, 1998). The range of 0.15 to 0.85 was considered acceptable (Nunnally, 1978). The Discrimination Index reflected the ability of each item to differentiate among participants with varying levels of ability. It was assessed using the Upper-Lower group method to calculate the Critical Ratio (CR). Participants were first divided into high- and low-performance groups based on total test scores. The top and bottom 27% were selected, and independent samples *t*-tests were conducted for each item to assess group differences. Items with CR values greater than 3.00 and significance levels of *p* < 0.05 were considered acceptable for inclusion (Lord, 2012) Reliability Analysis Internal consistency reliability was assessed using Cronbach’s alpha coefficient, with a coefficient value ≥0.70 considered acceptable and ≥0.80 indicating good reliability (Nunnally, 1978). Test–retest reliability was assessed using a two-way mixed-effects model, absolute consistency, and the intraclass correlation coefficient (ICC(3,1)) for single measurements. A subsample of 60 children (15 per age group), randomly selected from the four age groups, completed two assessments under the same conditions, with a two-week interval between assessments. Inter-rater reliability was assessed using a two-way random effects model, absolute agreement, and the average measurement ICC(2,k), where k = 2 raters, and 10% of participants were assessed simultaneously. The interpretation criteria for ICC values are as follows: <0.50 is poor, 0.50–0.75 is moderate, 0.75–0.90 is good, and >0.90 is excellent reliability (Wu, 2010; Fleiss & Cohen, 1973). The Kaiser-Meyer-Olkin (KMO) measure of sampling adequacy and Bartlett’s Test of Sphericity were performed to assess the suitability of the data for factor analysis. Exploratory Factor Analysis (EFA) was conducted using Maximum Likelihood (ML) extraction to extract the underlying factor structure. Confirmatory Factor Analysis (CFA) was then performed to test the structural validity of the MOBAK model. Items were treated as ordinal categorical variables, with model parameters estimated using the robust weighted least squares mean and variance adjusted (WLSMV) estimator (Muthén, 1997). When necessary, residual covariances are added between specific items based on theoretical grounds and modified indicators to improve model fit while maintaining theoretical consistency. All added residual correlations are between items within the same latent construct. Model fit is assessed comprehensively through multiple indices, including the chi-square-to-degrees-of-freedom ratio (χ^2^/df), Comparative Fit Index (CFI), Tucker–Lewis Index (TLI), Goodness-of-Fit Index (GFI), Root Mean Square Error of Approximation (RMSEA), and Standardized Residual Mean Square Root (SRMR). Model fit criteria are as follows: χ^2^/df < 3.0, CFI, TLI, GFI > 0.90, RMSEA, SRMR < 0.08 (Brown, 2019).

## 3. Results

### 3.1. MOBAK Difficulty and Discrimination Index

As can be seen from Table 3, in the MOBAK FMS test, Item Difficulty analysis. Object movement skills ranged from 0.51 to 0.59, while self-movement skills ranged from 0.57 to 0.67. The total score ranged from 0.54 to 0.63.

The Discrimination Index analysis at the dimensional level yielded highly significant results. The Critical Ratio (CR) values for the total ranged from 12.70 to 50.07, the object movement dimension ranged from 14.94 to 32.29, and for the self-movement dimension from 12.708 to 34.54, with all values reaching statistical significance (*p* < 0.001), as detailed in Table 4.

### 3.2. Reliability Testing of the MOBAK

#### 3.2.1. Internal Consistency Reliability

Homogeneity Reliability tests (which assume unidimensionality, as supported by EFA results) conducted on object movement skills, self-movement skills, and total test scores across different age groups and genders revealed reliability coefficients exceeding 0.8 for all age groups. Both object movement and self-movement skills, and the total test scores for children of different genders were above 0.8, in Table 5.

#### 3.2.2. Test–Retest Reliability

Test–retest reliability was assessed using the intraclass correlation coefficient (ICC) based on a two-way mixed-effects model with consistency type (ICC(3,1)), along with 95% confidence intervals (CIs). The results, summarized in Table 6, when testing was conducted at two-week intervals, the ICC values for all age groups were >0.9.

#### 3.2.3. Inter-Rater Reliability

Inter-rater reliability was based on a two-way random-effects model with consistency type for average measures (ICC(2,k)), as shown in Table 7. The ICC values for all age groups and subdomains were >0.9.

### 3.3. Structural Validity of the MOBAK

The results of the structural validity testing showed that the Kaiser-Meyer-Olkin (KMO) value was 0.838, the approximate chi-square value of Bartlett’s sphericity test was 548.395, and Bartlett’s test of sphericity indicated that the correlation matrix was significantly different from an identity matrix, supporting the factorability of the data. These findings indicate that the data were highly suitable for factor analysis, with a significance level of *p* < 0.01.

A total of 400 data points were extracted from each level for exploratory factor analysis, and the results showed that the total variance explained rate was 50.023%, which automatically reversed the two factors, namely, object movement and self-movement.

**Figure 1:**
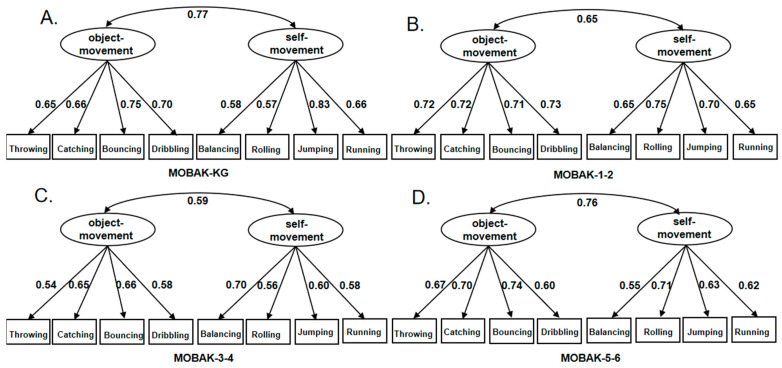
Confirmatory factor analysis (CFA) of the two-factor structure (object movement and self-movement) of the MOBAK test battery across four age groups.KG = Kindergarten (3–6 years); 1–2 = Grades 1–2 (6–8 years); 3–4 = Grades 3–4 (8–10 years); 5–6 = Grades 5–6 (10–12 years).

Subsequently, confirmatory factor analysis (CFA) was conducted to evaluate the model fit of the MOBAK assessment tool’s two-factor model across different age groups of children (Y. Li & Huang, 2007). The CFA results indicated that all fit indices met the ideal criteria: the Comparative Fit Index (CFI) was above 0.90, the Tucker–Lewis Index (TLI) was above 0.90, the Root Mean Square Error of Approximation (RMSEA) and the Standardized Root Mean Square Residual (SRMR) were below 0.08, see Table 8. Figure 1 illustrates the two-factor model of object movement and self-movement, including residual correlations between items within each factor. The factor loadings of the model for all age groups are higher than 0.5, indicating that the items are strongly correlated with the factors and that the overall model has a high degree of fit. The two-factor structure was strongly validated (Fornell & Larcker, 1981).

## 4. Discussion

### 4.1. MOBAK Item Difficulty and Discrimination in the Chinese Context

Item analysis revealed that the MOBAK items exhibited appropriate difficulty and strong discrimination—two distinct psychometric properties. Item difficulty (0.51–0.67) reflects how challenging each task is for the target population, ensuring the test avoids floor or ceiling effects. Item discrimination (CR values: 12.70–50.07, all *p* < 0.001) reflects the extent to which each task differentiates between children with higher and lower motor competence. Together, these findings indicate that the MOBAK items are both appropriately calibrated and effective at identifying individual differences in motor skills among Chinese children aged 3–12 years (Crocker & Algina, 1986). These findings are consistent with the robust discrimination reported in Western samples for standardized tools such as TGMD-3, collectively supporting the scientific validity of MOBAK as an effective assessment instrument (Ulrich, 1985).

The core strength of the MOBAK tool lies in its age-based structure, which tailors assessment modules to four developmental stages: preschool, grades 1–2, grades 3–4, and grades 5–6. Each stage includes progressively more challenging tasks aligned with children’s motor development, ranging from basic sensory-motor integration to complex, coordinated, and multi-task motor performance (Barnett et al., 2010; E. Herrmann et al., 2015). This aligns with the findings of Robinson et al. (2015) on the developmental trajectories of children’s FMS, consistent with the sequential nature of children’s motor skill development and adhering to the requirements of dynamic systems theory regarding the complexity and self-organizing characteristics of motor skills (Robinson et al., 2015; Gallahue, 2010; Goodway et al., 2019)

### 4.2. MOBAK System Reliability and Validity

Based on the methodological procedures for verifying the reliability and validity of assessment tools and the results of previous studies, a coefficient of >0.8 or the Cronbach’s Alpha of >0.7 for the Test–Retest Reliability, Inter-rater Reliability, and Internal Consistency Reliability could reflect ‘good’ reliability (Koo & Li, 2016; Chan, 2003; Streiner, 2003).

This study found that in the MOBAK tool test of Chinese children, the Test–Retest Reliability results showed the temporal stability of the MOBAK assessment tool, and the assessment results verified the measurement consistency of the MOBAK tool in measuring children’s FMS at different time points, further enhancing its reliability as a reliable assessment tool. Inter-rater Reliability further indicates that the MOBAK assessment tool has high operational objectivity among different scorers, with clear scoring criteria, ensuring the reliability of the assessment results. The Internal-Consistency Reliability of the MOBAK assessment tool shows high homogeneity, with Cronbach’s Alpha coefficients all exceeding 0.7, and some even exceeding 0.8, indicating a high degree of correlation between the assessment items under each dimension.

In terms of construct validity, this study verified the theoretical validity of the two-factor model (object movement and self movement) of the MOBAK assessment tool through exploratory factor analysis and confirmatory factor analysis. The exploratory factor analysis (EFA) extracted two common factors. clearly corresponding to object movement and self-movement. More importantly, CFA strongly supported the theoretical validity of this two-factor model, with excellent fit indices (CFI and TLI both greater than 0.90, and RMSEA all less than 0.08). The results of EFA and CFA not only validated the applicability of the MOBAK two-factor structure among Chinese children but, more critically, its factor structure and fit indices were highly consistent with findings from European and South American studies (C. Herrmann et al., 2015). This indicates that its core structure (object movement/self-movement) is robust in different cultural contexts (including Chinese culture) and can effectively distinguish and assess the FMS of children of different ages and genders, further consolidating its credibility as an international standardized tool. The high fit indices of CFA indicate that this structure can effectively capture the differentiation and integration characteristics of children’s basic abilities in a dynamic environment. Compared with other FMS assessment tools, MOBAK places more emphasis on multidimensional integrated skill assessment. By subdividing balancing, object movement, and self-movement, it can more accurately capture differences in children’s motor abilities (C. Herrmann et al., 2019a). Additionally, functional movement designs (e.g., ‘bouncing’) reduce cultural specificity interference, supporting its application in Chinese educational settings. This functional, outcome-oriented approach differs from process-focused assessments such as TGMD. TGMD emphasizes the execution quality of individual test movements (such as hip rotation during an overhead throw) without evaluating the achievement of the movement’s intended goal. In contrast, MOBAK’s design intrinsically links the movement to the successful attainment of a functional outcome (such as successfully hitting a target with a throw). This feature not only enhances the explanation of assessment results but also provides a scientific basis for personalized interventions in educational practice.

Beyond psychometric characteristics, the data from this study also offer insights into how cultural contexts influence the development of motor skills. This corroborates the core concept of ‘environmental constraints’ within dynamic systems theory.

The development of motor skills does not follow a universal, rigid sequence, but rather emerges as adaptive behaviour through repeated practice within the “opportunities” and ‘demands’ provided by specific cultural environments. Children’s motor ability characteristics are the product of continuous interaction between their individual potential and cultural environments (including physical education curricula, extracurricular activities, traditional games, etc.) (González-Víllora et al., 2019). Culturally distinctive physical activities encountered frequently in daily life, such as skipping rope, martial arts, gymnastics, and various forms of group games, profoundly shape their ‘repertoire of movement experiences.’ These experiences produced significant facilitation or transformation effects when interacting with the MOBAK test tasks, with self-movement generally performing well. This phenomenon is likely closely linked to the highly prevalent activity of skipping rope. Skipping rope is a quintessential task demanding rhythm, bodily coordination, and precise foot control. Its prolonged practice directly fortifies children’s abilities in dynamic balance, dual-foot take-off, and landing control. These abilities constitute precisely the core elements assessed in self-propulsion tasks such as “jumping” and “running” within the MOBAK test (Trecroci et al., 2015). Furthermore, fundamental martial arts stances like the “horse stance” and “bow stance” require practitioners to maintain a low centre of gravity and stability during static or slow movements, thereby significantly developing static balance, core strength, and limb proprioception. Meanwhile, martial arts forms incorporating rolls and evasive maneuvers directly correlate with the ‘rolling’ skill in MOBAK tasks, demanding keen spatial awareness and precise control over bodily positioning (B. Li et al., 2022). In contrast, the performance of Chinese children in certain “object movement” tasks (such as “throwing”) seems to be lower than the figures reported in the European samples (Cárcamo Oyarzún & Herrmann, 2020), which may reflect differences in sports experience. Although Chinese children engage in throwing activities, the frequency, form, and requirements of these activities in daily life and physical education lessons may differ from the Western movement patterns underlying the MOBAK test (such as overhand throws emphasizing power and accuracy). This necessitates a process of “translating” and “adapting” existing experiences, which may have impacted their immediate performance to some extent. The ingenuity of the MOBAK test battery lies in its successful capture and adaptation of these culturally shaped motor abilities through “function-oriented” rather than “skill-specific” task design. Rather than requiring children to demonstrate standardized “skipping rope” movements or “martial arts” routines, it extracts the fundamental motor elements common to these cultural activities—such as balance, coordination, rolling, and jumping—and translates them into neutral assessment tasks. For instance, MOBAK does not evaluate skipping rope technique, yet its “balancing” and “jumping” tasks effectively reflect the corresponding abilities enhanced through skipping practice. This design enables MOBAK to universally measure core motor competencies across children from diverse cultural backgrounds, free from the constraints of sport-specific technical details.

## 5. Conclusions and Limitations

### 5.1. Conclusions

Comprehensive psychometric evaluation demonstrated strong reliability, validity, and cross-cultural applicability of the MOBAK assessment tool for Chinese children. The results showed that it exhibited high levels of Internal Consistency reliability, test–retest reliability, Inter-rater Reliability, and structural validity in the assessment of object movement and self-movement skills across all age groups.

The present study extends this line of research by providing the first empirical evidence for the cultural validity of MOBAK in the Chinese context. It demonstrates good validity and reliability in the Chinese cultural context and is suitable for assessing the FMS and development of motor skills in children aged 3–12 years. It can serve as an effective tool for evaluating the development of FMS in Chinese children. The MOBAK battery’s two-factor structure corresponds to the curricular emphasis in the Physical Education and Health Curriculum Standards (2022 Edition) (An et al., 2022). It is recommended to develop a longitudinal tracking system based on the MOBAK module design to monitor the continuity of children’s skill development. By adopting MOBAK within school settings, educators can establish a longitudinal system for tracking motor skill development across different educational stages. Such continuity is particularly important in the Chinese context, where transitions between kindergarten and primary school often create discontinuities in physical education provision. A structured tracking system enables teachers to align learning objectives more effectively across stages and to design timely interventions that support children’s sustained motor development. This further enhances the scientific validity and effectiveness of the assessment.

### 5.2. Limitations

Firstly, cultural differences limit the applicability of assessment standards. Although MOBAK avoids culturally specific movements (such as baseball batting), there are significant differences between the daily physical training content of Chinese children (such as skipping rope and martial arts) and that of Western children, which may result in some assessment indicators (such as the ‘throwing’ distance standard) not aligning with the actual abilities of Chinese children.

Moreover, China’s prevalent nuclear family structure, coupled with substantial disparities in educational resources between urban and rural areas as well as across regions, alongside a cultural environment that places high emphasis on academic achievement, may collectively shape the unique trajectory and pace of motor skill development among Chinese children. These structural differences in developmental mechanisms suggest that motor development in Chinese children may not fully align with theoretical models established using Western samples. Future research necessitates incorporating these socio-ecological variables to conduct more in-depth mechanistic investigations. This study did not examine the influence of social demographic variables. It is suggested that future research adopt the MIMIC model to systematically evaluate the effects of age, gender, BMI, and other relevant covariates on MOBAK performance.

## Figures and Tables

**Table 1:** Participant characteristics by age group and sex (M ± SD).

Sex	MOBAK KG		MOBAK1–2		MOBAK3–4		MOBAK5–6	
	*n*	Age	*n*	Age	*n*	Age	*n*	Age
Girls	275	4.93 ± 0.92	102	7.18 ± 0.66	102	9.22 ± 0.60	98	11.16 ± 0.75
Boys	325	4.71 ± 1.04	98	7.16 ± 0.71	98	9.26 ± 0.59	102	11.22 ± 0.63
Total	600	4.80 ± 0.99	200	7.17 ± 0.68	200	9.23 ± 0.60	200	11.20 ± 0.70

Note: KG = Kindergarten (3–6 years); 1–2 = Grades 1–2 (6–8 years); 3–4 = Grades 3–4 (8–10 years); 5–6 = Grades 5–6 (10–12 years).

**Table 2:** Descriptive summary of the items in the MOBAK Battery for each test instrument (in detail (Cárcamo Oyarzún & Herrmann, 2020; C. Herrmann et al., 2019b).

Test Item	Level	Description
Object movement		
Throwing	KG	The child throws six juggling balls from a 1.5 m distance at a target
	1–2	The child throws six juggling balls from a 2.0 m distance at a target.
	3–4	The child throws six juggling balls from a 3.0 m distance at a target.
	5–6	The child throws six juggling balls from a 3.5 m distance at a target.
Catching	KG	The test leader drops a small basketball from 2 m to the ground so that the ball bounces at least 1.3 m above the ground. The child catches the ball after the reversal point.
	1–2	The test leader causes a rubber ball to fall to the ground in an accelerated manner, causing the ball to jump up to at least 1.3 m. The child catches the ball after the turning point.
	3–4	The child throws up a ball and catches it behind a line at a 1.5 m distance.
	5–6	The child throws a tennis ball at a wall from a scratch line at a distance of 3.0 m. The child catches the tennis ball directly from the air when it bounces back.
Bouncing	KG	The child bounces a volleyball on the ground with both hands at least five times in a row and catches it again without losing the ball.
	1–2	The child bounces a small basketball (size 3) through a marked corridor (5.0 × 1.0 m) without losing the ball.
	3–4	The child bounces a small basketball (size 3) back and forth through a marked corridor (7.5 × 1.4 m) with obstacles, without losing the ball.
	5–6	The child bounces a basketball (size 6) back and forth through a marked corridor (8.0 × 1.1 m) with four obstacles of 0.7 m width, without losing the ball.
Dribbling	KG	The child dribbles a futsal ball through a marked corridor (9.0 × 2.8 m) with two obstacles of 1.5 m width, without losing the ball.
	1–2	The child dribbles a futsal ball (size 4) back through a marked corridor (5.0 × 1.0 m) without losing the ball.
	3–4	The child dribbles a futsal ball (size 4) back and forth through a marked corridor (7.5 × 1.4 m) with obstacles without losing the ball.
	5–6	The child dribbles a futsal ball (size 4) back and forth through a marked corridor (8.0 × 1.1 m) with four obstacles of 0.7 m width, without losing the ball.
Self Movement		
Balancing	KG	The child walks forwards and backwards over an overturned long bench.
	1–2	The child balances over an overturned long bench placed on a springboard (forming a see-saw) without leaving it.
	3–4	The child balances back and forth across a long upside-down bench with two boxes attached that have to be overstepped. No follow-up steps.
	5–6	The child balances back and forth over an overturned long bench placed on a springboard, passing two obstacles taped to the bench (L: 17 cm, W: 10 cm, H: 12 cm) without touching them.
Rolling	KG	The child performs a fluent forward roll down an inclined mat and lands on his feet.
	1–2	The child performs a fluent forward roll on a mat track.
	3–4	The child performs a fluent forward roll, starting with a jump onto a pair of vaulting boxes.
	5–6	The child performs a forward roll, starting with a jump over a set-up banana box.
Jumping	KG	The child jumps a distance of 1.5 m on one foot, turns around, and jumps back 1.5 m on the other foot.
	1–2	The child jumps fluently over four carpet tiles (0.35 × 0.35) at a distance of 0.4 m each. Between the tiles, one-legged, beside the tiles, with straddled legs.
	3–4	The child skips rope continuously in place for 20 s
	5–6	The child skips rope in place for 20 s, changing rhythm after 10 s.
Running	KG	The child jumps a distance of 1.5 m on one foot, turns around, and jumps back 1.5 m on the other foot.
	1–2	The child moves back and forth twice on a 3.0 m long ground mark, performing sidesteps.
	3–4	The child moves forward and sideways along a figure eight (2.0 m × 4.0 m) marked on the floor.
	5–6	The child moves forward and sideways along a figure eight (4.0 × 4.0 m) marked on the floor. In running forward, the child jumps through three evenly spaced hoops lying on the floor.

Note: KG = Kindergarten (3–6 years); 1–2 = Grades 1–2 (6–8 years); 3–4 = Grades 3–4 (8–10 years); 5–6 = Grades 5–6 (10–12 years).

**Table 3:** Item difficulty indices across age groups for fundamental motor skills assessment.

Age Group	Object Movement	Self-Movement	Total
MOBAK—KG	0.59	0.67	0.63
MOBAK—1–2	0.58	0.65	0.61
MOBAK—3–4	0.55	0.67	0.61
MOBAK—5–6	0.51	0.57	0.54

Note: KG = Kindergarten (3–6 years); 1–2 = Grades 1–2 (6–8 years); 3–4 = Grades 3–4 (8–10 years); 5–6 = Grades 5–6 (10–12 years).

**Table 4:** Mean comparison of scores between the high-score group and the low-score group for each item.

Age Group	Dimension Total	Group (Mean ± SD)		Effect Size	Significance
		Low Group Bottom 27% (*n* = 188)	High Group Top 27% (*n* = 190)	CR	*p*
MOBAK—KG	Object movement	2.22 ± 1.62	6.75 ± 1.05	32.29	<0.001 **
	Self-movement	3.24 ± 1.38	7.36 ± 0.88	34.54	<0.001 **
	Total	5.46 ± 2.04	14.12 ± 1.03	50.07	<0.001 **
MOBAK—1–2	Object movement	2.76 ± 0.84	6.88 ± 1.33	20.923	<0.001 **
	Self-movement	3.53 ± 0.68	6.97 ± 1.18	20.306	<0.001 **
	Total	6.29 ± 0.98	13.85 ± 1.56	32.736	<0.001 **
MOBAK—3–4	Object movement	2.33 ± 1.19	6.62 ± 1.03	21.826	<0.001 **
	Self-movement	4.33 ± 0.93	6.77 ± 1.21	12.708	<0.001 **
	Total	6.66 ± 1.33	13.38 ± 1.32	28.90	<0.001 **
MOBAK—5–6	Object movement	2.41 ± 1.26	5.64 ± 1.33	14.948	<0.001 **
	Self-movement	3.19 ± 1.15	5.97 ± 1.17	14.415	<0.001 **
	Total	5.60 ± 1.68	11.61 ± 1.81	20.660	<0.001 **

Note: KG = Kindergarten (3–6 years); 1–2 = Grades 1–2 (6–8 years); 3–4 = Grades 3–4 (8–10 years); 5–6 = Grades 5–6 (10–12 years). ** *p* < 0.01.

**Table 5:** Internal Consistency Reliability Results of the MOBAK Test Across Age Groups.

Age Group	Object Movement	Self-Movement	Total Score
MOBAK—KG	0.882	0.868	0.921
MOBAK—1–2	0.895	0.879	0.921
MOBAK—3–4	0.840	0.840	0.889
MOBAK—5–6	0.875	0.849	0.915
Average	0.873	0.859	0.911
Total number	0.870	0.865	0.915
Girls	0.859	0.865	0.919
Boys	0.875	0.865	0.919

Note: KG = Kindergarten (3–6 years); 1–2 = Grades 1–2 (6–8 years); 3–4 = Grades 3–4 (8–10 years); 5–6 = Grades 5–6 (10–12 years).

**Table 6:** Test–retest reliability using ICC(3,1) with 95% confidence intervals.

Age Group	Object Movement (95% CI)	Self-Movement (95% CI)	Total Score (95% CI)
MOBAK-KG	0.905 [0.846–0.942]	0.942 [0.905–0.965]	0.945 [0.910–0.967]
MOBAK-1–2	0.929 [0.883–0.957]	0.906 [0.847–0.943]	0.940 [0.901–0.963]
MOBAK-3–4	0.919 [0.869–0.951]	0.867 [0.787–0.918]	0.917 [0.865–0.950]
MOBAK-5–6	0.903 [0.842–0.941]	0.903 [0.843–0.941]	0.922 [0.873–0.953]

ICC interpretation: <0.50 poor, 0.50–0.75 moderate, 0.75–0.90 good, >0.90 excellent. Note: KG = Kindergarten (3–6 years); 1–2 = Grades 1–2 (6–8 years); 3–4 = Grades 3–4 (8–10 years); 5–6 = Grades 5–6 (10–12 years).

**Table 7:** Inter-rater reliability using ICC(2,k) with 95% confidence intervals.

Age Group	Object Movement (95% CI)	Self-Movement (95% CI)	Total Score (95% CI)
MOBAK-KG	0.942 [0.903–0.965]	0.943 [0.905–0.966]	0.957 [0.928–0.974]
MOBAK-1–2	0.943 [0.904–0.966]	0.945 [0.907–0.967]	0.955 [0.925–0.973]
MOBAK-3–4	0.947 [0.911–0.968]	0.917 [0.861–0.950]	0.947 [0.911–0.968]
MOBAK-5–6	0.933 [0.888–0.960]	0.921 [0.868–0.953]	0.947 [0.911–0.968]

ICC Interpretation: <0.50 Poor, 0.50–0.75 Moderate, 0.75–0.90 Good, >0.90 Excellent. Note: KG = Kindergarten (3–6 years); 1–2 = Grades 1–2 (6–8 years); 3–4 = Grades 3–4 (8–10 years); 5–6 = Grades 5–6 (10–12 years).

**Table 8:** Overall Fit Coefficients Table.

Age Group	χ^2^/df	CFI	TLI	GFI	RMSEA	SRMR
MOBAK—KG	2.968	0.977	0.964	0.978	0.057	0.034
MOBAK—1–2	2.013	0.964	0.946	0.956	0.071	0.040
MOBAK—3–4	1.431	0.972	0.959	0.968	0.047	0.044
MOBAK—5–6	1.452	0.980	0.970	0.967	0.048	0.037
Acceptable fit criteria	<3.0	>0.90	>0.90	>0.90	<0.08	<0.08

Note: KG = Kindergarten (3–6 years); 1–2 = Grades 1–2 (6–8 years); 3–4 = Grades 3–4 (8–10 years); 5–6 = Grades 5–6 (10–12 years). CFI comparative fit index, TLI Tucker–Lewis index, GFI goodness-of-fit index, RMSEA root mean square error of approximation, SRMR standardized root mean square residual. Model estimation using the weighted least squares mean and variance adjusted (WLSMV) method.

## Data Availability

The data that support the findings of this study are available from the corresponding author upon reasonable request.
